# Sometimes the Same, Sometimes Different: Understanding Self-Assembly Algorithms in Coordination Networks

**DOI:** 10.3390/polym10121369

**Published:** 2018-12-11

**Authors:** Y. Maximilian Klein, Alessandro Prescimone, Mariia Karpacheva, Edwin C. Constable, Catherine E. Housecroft

**Affiliations:** Department of Chemistry, University of Basel, BPR 1096, Mattenstrasse 24a, 4058 Basel, Switzerland; maximilian.klein@psi.ch (Y.M.K.); Alessandro.prescimone@unibas.ch (A.P.); mariia.karpacheva@unibas.ch (M.K.); edwin.constable@unibas.ch (E.C.C.)

**Keywords:** tetratopic ligands, 4,2′:6′,4″-terpyridine, 3,2′:6′,3″-terpyridine, metal-organic framework, cobalt(II) thiocyanate

## Abstract

The syntheses and characterizations of three new ligands containing two 4,2′:6′,4″-tpy or two 3,2′:6′,3″-tpy metal-binding domains are reported. The ligands possess different alkyloxy functionalities attached to the central phenylene spacer: *n*-pentyloxy in **3**, 4-phenyl-*n*-butoxy in **4**, benzyloxy in **5**. Crystal growth under ambient conditions has led to the formation of {[Co(NCS)_2_(**3**)]·0.8C_6_H_4_Cl_2_}*_n_* and {[Co(NCS)_2_(**4**)]·1.6H_2_O·1.2C_6_H_4_Cl_2_}*_n_*, with structures confirmed by single crystal X-ray diffraction. Both the cobalt(II) center and ligand **3** or **4** act as 4-connecting nodes and both {[Co(NCS)_2_(**3**)]·0.8C_6_H_4_Cl_2_}*_n_* and {[Co(NCS)_2_(**4**)]·1.6H_2_O·1.2C_6_H_4_Cl_2_}*_n_* possess a 3D *cds* net despite the fact that **3** and **4** contain two 4,2′:6′,4″-tpy and two 3,2′:6′,3″-tpy units, respectively. Taken in conjunction with previously reported data, the results indicate that the role of the alkyloxy substituent is more significant than the choice of 4,2′:6′,4″- or 3,2′:6′,3″-tpy isomer in determining the assembly of a particular 3D net. The combination of Co(NCS)_2_ with **5** resulted in the formation of the discrete molecular complex [Co(NCS)_2_(MeOH)_2_(**5**)_2_]·2CHCl_3_·2MeOH in which **5** acts as a monodentate ligand. The pendant phenyls and both coordinated and non-coordinated 4,2′:6′,4″-tpy units are involved in efficient π-stacking interactions.

## 1. Introduction

**Scheme 1:**
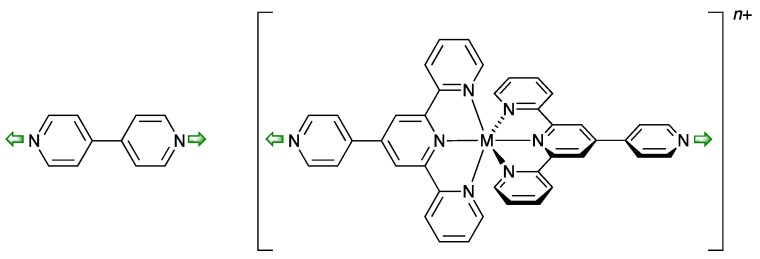
An [M(tpy)_2_]*^n^*^+^ complex is considered as an expanded 4,4′-bpy.

**Scheme 2:**
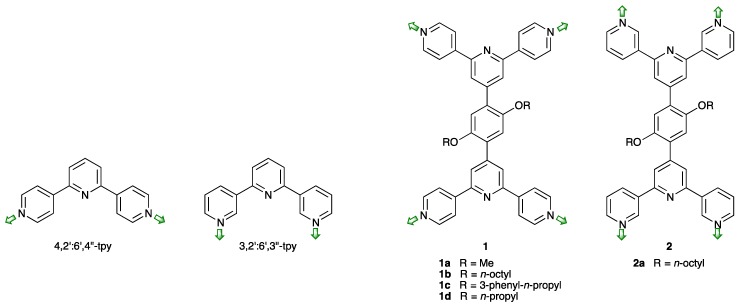
Structures of 4,2′:6′,4″- and 3,2′:6′,3″-tpy of general ligand types **1** (with 4,2′:6′,4″-tpy units) and **2** (with 3,2′:6′,3″-tpy units), and structures of ligands **1a**–**1d** and **2a**. The green arrows show the vectoral orientation of the lone pairs involved in formation of the coordination network.

Polymeric materials are essential to mankind, both as the crucial biomolecules enabling life processes and as the materials at the core of our socioeconomic structures. Metallopolymers are an important subset of polymers, in which the incorporation of metal centers allows the blending of new properties which are not readily accessed through polymeric structures based only on C, H, N, O, S and P atoms. An important class of metallopolymers are the coordination polymers and networks, in which the metal centers are incorporated through coordination rather than covalent bonds. Specifically, coordination polymers and networks are infinite assemblies in which the metal centers are connected by coordinated organic molecules. The coordination number and coordination geometry of the metal centers provide an exquisite control over the dimensionality as well as the topographical and topological features of the coordination assemblies. Ligands with pyridine nitrogen donors comprise some of the most commonly utilized linkers, for example [1,2,3,4,5,6,7], with 4,4′-bipyridine (4,4′-bpy, Scheme 1) used extensively as a rigid organic linker in coordination assemblies. An [M(pytpy)_2_]*^n^*^+^ (pytpy = 4′-(4-pyridyl)-2,2′:6′,2″-terpyridine) is an expanded 4,4′-bpy since both can bind two metal ions and possess similar vectorial properties (Scheme 1) [8]. Newkome and coworkers have demonstrated the assembly of metallocages based upon [M(tpy)_2_]*^n^*^+^ building blocks with other vectorial properties [9]. By changing from the 2,2′:6′,2″-isomer of terpyridine to either 3,2′:6′,3″- or 4,2′:6′,4″-terpyridine (3,2′:6′,3″- or 4,2′:6′,4″-tpy, Scheme 2) it should be possible to modify the consequences of the coordination interactions on the network assembly in a controlled and predictable manner. The ligands 3,2′:6′,3″- or 4,2′:6′,4″-tpy both bind metal ions only through the four outer *N*-donors and there is no literature precedent for metal-coordination through the central pyridine ring. We and others have made widespread use of 4,2′:6′,4″-tpy ligands, typically functionalized in the 4′-position for the assembly of 1D-coordination polymers and 2D-networks [10,11,12]. Examples of 3D-architectures incorporating 4,2′:6′,4″-tpy ligands are rare [13] and only a very few examples of 2D- and 3D-assemblies directed by 3,2′:6′,3″-tpy ligands have been reported [14,15,16,17,18,19,20]. The most significant difference between a 4,2′:6′,4″-tpy and 3,2′:6′,3″-tpy is the effect that inter-ring C–C bond rotation has on the mutual directionality of the outer nitrogen donors. For 4,2′:6′,4″-tpy, there is no change in the vectorial orientation of the nitrogen donors, while for 3,2′:6′,3″-tpy there is significant variation with the interannular bond rotation [11]. This in turn increases the structural diversity to be expected with 3,2′:6′,3″-tpy, at the expense of the predictability of the self-assembly algorithm. In Scheme 2, the 3,2′:6′,3″-tpy unit is drawn in an orientation preorganized for the formation of metallomacrocycles, as observed with 1-(3,2′:6′,3′′-terpyridin-4′-yl)ferrocene [21] and 5-(3,2′:6′,3′′-terpyridin-4′-yl)-*N,N*-diphenylthiophen- 2-amine [22].

Covalent linkage of two 4,2′:6′,4″- or 3,2′:6′,3″-tpy domains combines two ditopic metal-binding domains into a tetratopic ligand as exemplified with the general ligands **1** and **2** in Scheme 2. We have developed a suite of such ligands, typically with 1,4-phenylene spacers functionalized with alkyloxy groups to increase solubility in organic solvents [23]. The nature of the OR group is critical in determining the outcome of the self-assembly. For example, the reaction of **1a** or **1b** (Scheme 2) with zinc(II) halides leads to (4,4) nets in which **1a** or **1b** is the 4-connecting node. However, in the case of **1a**, simple 2D-sheets are observed, whereas the presence of the longer *n*-octyl groups in **1b** leads to 2D→2D parallel interpenetration with the alkyloxy chains in extended conformations and directed within each corrugated sheet [23,24]. We have also demonstrated that introducing a pendant aryl group can facilitate face-to-face π-interactions within the solid-state assembly. In the case of [Zn_2_Br_4_(**1c**)**^.^**H_2_O]*_n_* (see Scheme 2 for **1c**), 2-fold interpenetrating *nbo* nets are observed with inter-net π-stacking resulting in tightly associated interpenetrated nets and, as a consequence, large void spaces in the lattice [25].

**Scheme 3:**
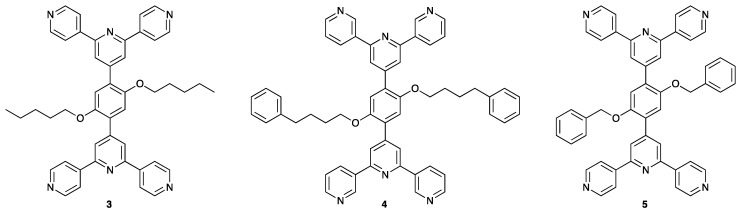
Structures of ligands **3**–**5.**

In this paper, we describe reactions of ligands **3**–**5** (Scheme 3) with Co(NCS)_2_. Upon interacting with pyridine donors, Co(NCS)_2_ typically generates octahedral cobalt(II) centers with *trans*-thiocyanato ligands and four pyridine nitrogen donors defining the equatorial plane. It is therefore expected to act as a 4-connecting node giving access to a range of different networks [26,27]. We have shown that the reaction of Co(NCS)_2_ with **1d** (Scheme 2) leads to a 3D *cds* net in which both metal and ligand are 4-connecting nodes [28]. Increasing the length of the alkyloxy chain to R = *n*-octyl and replacing the 4,2′:6′,4″- by a 3,2′:6′,3″-tpy (**2a**, Scheme 2) switches the 3D assembly from a *cds* to an *lvt* net [20]. In the present work, **3** was selected in order to investigate the effect of lengthening the alkyloxy chain from three to five carbon atoms—would the *cds* net be retained? Ligand **4** is comparable with **2a** in terms of the 3,2′:6′,3″-tpy donor set but introduces terminal phenyl domains in the alkyloxy substituents. Both **3** and **5** possess 4,2′:6′,4″-tpy units, but the inclusion of the pendant phenyls in **5** was expected to affect the assembly by enabling arene–arene π-interactions.

## 2. Materials and Methods

### 2.1. General

^1^H and ^13^C NMR spectra were recorded on a Bruker DRX-500 NMR spectrometer (Bruker BioSpin AG, Fällanden, Switzerland) with chemical shifts referenced to residual solvent peaks (TMS = *δ* 0 ppm). Electrospray ionization (ESI) mass spectra were measured on a Shimazu LCMS 2020 instrument (Shimadzu Schweiz GmbH, Roemerstr., Switzerland) and high resolution ESI (HR-ESI) mass spectra on a Bruker maXis 4G QTOF instrument (Bruker BioSpin AG, Fällanden, Switzerland); samples were introduced as MeCN solutions containing 0.1% formic acid or 0.1% trifluoroacetic acid (TFA) for electrospray mass spectrometry (ESI MS) and high-resolution ESI MS, respectively. Commercially available precursors were purchased from Fluorochem (Hadfield, UK), Sigma-Aldrich (Buchs SG, Switzerland), TCI (Eschborn, Germany) or Acros (Chemie Brunschwig AG, Basel, Switzerland) and used without further purification. Compound **4b** was prepared following the literature and NMR spectroscopic data were in accord with those reported [29].

### 2.2. Compound ***3a***

Compound **3a** was prepared by adapting a literature method [29]. 2,5-Dibromohydroquinone (2.0 g, 7.47 mmol), 1-bromopentane (2.36 mL, 2.88 g, 18.7 mmol) and anhydrous K_2_CO_3_ (3.1 g, 22.4 mmol) were dissolved in dry DMF (100 mL) and the mixture was heated at 100 °C for ca. 15 h. The reaction mixture was cooled to room temperature, added to a beaker containing 100 mL of ice-water and stirred for 30 min. The precipitate that formed was separated by filtration and washed with water (3 × 30 mL) and dried in vacuo. Compound **3a** was isolated as a white powder (2.85 g, 6.98 mmol, 93.5%). ^1^H NMR (500 MHz, CDCl_3_) *δ*/ppm 7.09 (s, 2H, H^C3^), 3.95 (t, *J* = 6.5 Hz, 4H, H^a^), 1.81 (m, 4H, H^b^), 1.46 (m, 4H, H^c^), 1.39 (m, 4H, H^d^), 0.94 (t, *J* = 7.2 Hz, 6H). ^13^C{^1^H} NMR (126 MHz, CDCl_3_) *δ*/ppm 150.2 (C^C2^), 118.6 (C^C3^), 111.3 (C^C1^), 70.5 (C^a^), 29.0 (C^b^), 28.3 (C^c^), 22.5 (C^d^), 14.2 (C^e^). In reference [29], ^1^H NMR signals were unassigned, and ^13^C NMR spectroscopic data were not reported. 

### 2.3. Compound ***4a***

Compound **4a** has been reported but we find the following method more convenient. The method was as for **3a** starting with 2,5-dibromohydroquinone (1.5 g, 5.6 mmol), 1-bromo-4-phenylbutane (0.96 mL, 3.04 g, 14 mmol) and anhydrous K_2_CO_3_ (2.32 g, 16.8 mmol) in dry DMF (100 mL). Then, **4a** was isolated as a white powder (2.67 g, 5.02 mmol, 89.6%). The NMR spectroscopic data agreed with those published [29].

### 2.4. Compound ***5a***

Compound **5a** has previously been reported [30], but we find the following synthesis more convenient. The method was as for **3a** starting with 2,5-dibromohydroquinone (1.5 g, 5.6 mmol), benzyl chloride (1.61 mL, 1.77 g, 14.0 mmol) and anhydrous K_2_CO_3_ (2.32 g, 16.8 mmol) in dry DMF (100 mL). Then, **5a** was obtained as a white powder (2.19 g, 4.89 mmol, 87.3%). ^1^H NMR spectroscopic data were in accord with those reported [30]. ^13^C{^1^H} NMR (126 MHz, CDCl_3_) *δ*/ppm 150.2 (C^C2c^), 136.3 (C^D1^), 128.8 (C^D3^), 128.3 (C^D4^), 127.4 (C^D2^), 119.4 (C^C3^), 111.7 (C^C1^), 72.1 (C^a^). 

### 2.5. Compound ***3b***

Compound **3a** (1.8 g, 4.41 mmol) and dry Et_2_O (150 mL) were added to a dried flask and cooled to 0 °C in an ice bath. *^n^*BuLi solution (1.6 M in hexanes, 8.27 mL, 13.2 mmol) was slowly added to the solution of **3a** over 20 min and the temperature maintained at 0 °C for 6 h. Dry DMF (1.02 mL, 13.2 mmol) was then added and the solution stirred for 15 h, while warming up to room temperature. The reaction was neutralized with saturated aqueous NH_4_Cl solution and extracted with CH_2_Cl_2_ (200 mL). The organic phase was dried over MgSO_4_ and concentrated in vacuo. Compound **3b** was obtained as a yellow solid (1.14 g, 3.72 mmol, 84.4%) and used without further purification. ^1^H NMR (500 MHz, CDCl_3_) *δ*/ppm 10.51 (s, 2H, H^CHO^), 7.42 (s, 2H, H^C3^), 4.08 (t, *J* = 6.5 Hz, 4H, H^a^), 1.87–1.76 (m, 4H, H^b^), 1.52–1.31 (m, 8H, H^c+d^), 0.93 (t, *J* = 7.2 Hz, 6H, H^e^). ^13^C{^1^H} NMR (126 MHz, CDCl_3_) *δ*/ppm 189.6 (C^CHO^), 155.3 (C^C2^), 129.4 (C^C1^), 111.7 (C^C3^), 69.3 (C^a^), 28.8 (C^b^), 28.3 (C^c^), 22.5 (C^d^), 14.1 (C^e^).

### 2.6. Compound ***5b***

The method was the same as for **3b** but starting with **5a** (2.0 g, 4.46 mmol) and *^n^*BuLi (1.6 M in hexanes, 8.36 mL, 13.4 mmol). Compound **2b** was obtained as a yellow solid (1.23 g, 3.55 mmol, 79.6%) and was used without further purification. ^1^H NMR spectroscopic data matched those reported [29]. ^13^C{^1^H} NMR (126 MHz, CDCl_3_) *δ*/ppm 189.2 (C^CHO^), 155.2 (C^C2^), 135.8 (C^D1^), 129.7 (C^C1^), 128.9 (C^D2/D3^), 128.5 (C^D4^), 127.7 (C^D2/D3^), 112.5 (C^C3^), 71.3 (C^a^).

### 2.7. Compound ***3***

Compound **3b** (0.3 g, 0.98 mmol) was dissolved in EtOH (100 mL), then 4-acetylpyridine (0.45 mL, 0.49 g, 3.92 mmol) and crushed solid KOH (0.22 g, 3.92 mmol) were added in one portion. Aqueous NH_3_ (32%, 7.8 mL) was added dropwise and the reaction mixture was stirred at room temperature for ca. 15 h. The precipitate that had formed was collected by filtration and washed with water, EtOH and Et_2_O (3 × 10 mL, each). Compound **3** was obtained as a white solid (0.13 g, 0.18 mmol, 18.8%). M.p. = 297 °C. ^1^H NMR (500 MHz, CDCl_3_) δ*/*ppm 8.81 (m, 8H, H^A2^), 8.15–8.07 (m, 12H, H^B3+A3^), 7.16 (s, 2H, H^C3^), 4.07 (t, *J* = 6.3 Hz, 4H, H^a^), 1.87 (m, 4H, H^b^), 1.37 (m, 4H, H^c^), 1.32–1.19 (m, 4H, H^d^), 0.77 (t, *J* = 7.3 Hz, 6H, H^e^). ^13^C{^1^H} NMR (126 MHz, CDCl_3_) δ*/*ppm 154.8 (C^B2^), 150.7 (C^C2^), 150.6 (C^A2^), 148.4 (C^B4^), 146.4 (C^A4^), 129.2 (C^C1^), 121.7 (C^B3^), 121.3 (C^A3^), 115.3 (C^C3^), 69.9 (C^a^), 29.27 (C^b^), 28.61 (C^c^), 22.53 (C^d^), 14.02 (C^e^). ESI-MS *m*/*z* 754.40 [M + H + MeCN]^+^ (base peak, calc. 754.39), 713.35 [M + H]^+^ (calc. 713.36). HR ESI-MS *m*/*z* 713.3600 [M + H]^+^ (calc. 713.3599).

### 2.8. Compound ***4***

The method was as for compound **3** but starting with **4b** (0.69 g, 1.62 mmol) and 3-acetylpyridine (0.82 mL, 0.89 g, 7.25 mmol), crushed KOH (0.41 g, 7.25 mmol) and aqueous NH_3_ (32%, 12.4 mL). Compound **4** was isolated as a white solid (0.47 g, 0.56 mmol, 34.7%). M.p. = 253 °C. ^1^H NMR (500 MHz, CDCl_3_) δ*/*ppm 9.38 (d, *J* = 2.2 Hz, 4H, H^A2^), 8.73 (m, 4H, H^A6^), 8.50 (dd, *J* = 7.9, 2.0 Hz, 4H, H^A4^), 8.01 (s, 4H, H^B3^), 7.45 (dd, *J* = 8.0, 7.9 Hz, 4H, H^A5^), 7.17–7.12 (m, 6H, H^C3+D3^), 7.09 (m, 2H, H^D4^), 6.98 (m, 4H, H^D2^), 4.07 (t, *J* = 6.1 Hz, 4H, H^a^), 2.55 (t, *J* = 7.5 Hz, 4H, H^d^), 1.87–1.78 (m, 4H, H^b^), 1.75–1.67 (m, 4H, H^c^). ^13^C{^1^H} NMR (126 MHz, CDCl_3_) δ*/*ppm 154.9 (C^B2^), 150.7 (C^C2^), 150.4 (C^A6^), 148.6 (C^A2^), 148.1 (C^B4^), 141.9 (C^D1^), 134.8 (C^A3^), 134.6 (C^A4^), 129.5 (C^C1^), 128.45 (C^D3^), 128.4 (C^D2^), 125.9 (C^D4^), 123.8 (C^A5^), 120.3 (C^B3^), 115.5 (C^C3^), 69.7 (C^a^), 35.5 (C^d^), 29.1 (C^b^), 28.0 (C^c^). ESI-MS *m*/*z* 837.40 [M + H]^+^ (base peak, calc. 837.39). HR ESI-MS *m*/*z* 837.3901 [M + H]^+^ (calc. 837.3912).

### 2.9. Compound ***5***

The method was as for **3**, starting with **5b** (1.06 g, 3.06 mmol), 4-acetylpyridine (1.56 mL, 1.7 g, 13.8 mmol), crushed KOH (0.77 g, 13.8 mmol), and aqueous NH_3_ (32%, 24 mL). Compound **5** was isolated as a white solid (0.87 g, 1.16 mmol, 37.8%). Decomp. > 280 °C. ^1^H NMR (500 MHz, CDCl_3_) δ*/*ppm 8.77 (m, 8H, H^A2^), 8.07 (s, 4H, H^B3^), 7.96 (m, 8H, H^A3^), 7.40–7.33 (m, 10H, H^D2+D3+D4^), 7.31 (s, 2H, H^C3^), 5.19 (s, 4H, H^a^). ^13^C{^1^H} NMR (126 MHz, CDCl_3_) δ*/*ppm 154.9 (C^B2^), 150.7 (C^C2^), 150.5 (C^A2^), 147.7 (C^B4^), 146.3 (C^A4^), 136.2 (C^D1^), 129.5 (C^C1^), 129.0 (C^D3^), 128.7 (C^D4^), 127.9 (C^D2^), 121.5 (C^B3^), 121.4 (C^A3^), 116.1 (C^C3^), 72.1 (C^a^). ESI-MS *m*/*z* 794.30 [M + H + MeCN]^+^ (calc. 794.32), 753.35 [M + H]^+^ (calc. 753.30). HR ESI-MS *m*/*z* 753.2967 [M + H]^+^ (calc. 753.2973).

### 2.10. {[Co(NCS)_2_(3)]·0.8C_6_H_4_Cl_2_}_n_

A solution of Co(NCS)_2_ (1.75 mg, 0.01 mmol) in MeOH (8 mL) was layered over a solution of **3** (7.13 mg, 0.01 mmol) in 1,2-dichlorobenzene (5 mL). A few pink crystals of [Co(NCS)_2_(**3**)·0.8C_6_H_4_Cl_2_]*_n_* were obtained after 2–4 weeks. Insufficient material was obtained for powder X-ray diffraction.

### 2.11. {[Co(NCS)_2_(4)]·1.6H_2_O·1.2C_6_H_4_Cl_2_}_n_

A solution of Co(NCS)_2_ (3.5 mg, 0.02 mmol) in MeOH (8 mL) was layered over a solution of **4** (8.37 mg, 0.01 mmol) in 1,2-dichlorobenzene (5 mL). Pink crystals of [Co(NCS)_2_(**4**)·1.6H_2_O·1.2C_6_H_4_Cl_2_]*_n_* (2.2 mg, 0.0018 mmol, 18% based on **5**) were obtained after 2–4 weeks. The bulk sample was characterized by powder X-ray diffraction (Figure S1).

### 2.12. [Co(NCS)_2_(MeOH)_2_(5)_2_]·2CHCl_3_·2MeOH

A solution of Co(NCS)_2_ (3.5 mg, 0.02 mmol) in MeOH (8 mL) was layered over a solution of **5** (15.1 mg, 0.02 mmol) in CHCl_3_ (5 mL). A small crop of pink crystals of [Co(NCS)_2_(MeOH)_2_(**5**)_2_·2CHCl_3_·2MeOH] was obtained after 2–4 weeks. Insufficient material was obtained for powder X-ray diffraction

### 2.13. Crystallography

Single crystal data were collected on a Bruker APEX-II diffractometer (Bruker Biospin AG, Fällanden, Switzerland); data reduction, solution and refinement used APEX2, SuperFlip and CRYSTALS respectively [31,32,33]. Structure analysis used Mercury v. 3.10 [34,35]. In {[Co(NCS)_2_(**3**)]·0.8C_6_H_4_Cl_2_}*_n_*, the dichlorobenzene molecule was refined as rigid body (occupancy = 0.8 per formula unit). In addition, the aliphatic chain showed high thermal motion, and was refined isotropically using chemically reasonable restraints to bond distances and angles. In {[Co(NCS)_2_(**4**)]·1.6H_2_O·1.2C_6_H_4_Cl_2_}*_n_*, the side chain of the organic ligand is disordered with high thermal motion; as a result, the phenyl ring was refined with rigid body restraints and the whole chain was refined isotropically. Powder diffraction data were collected on a Stoe Stadi P powder diffractometer (Stoe & Cie GmbH, Darmstadt, Germany).

{[Co(NCS)_2_(**3**)]·0.8C_6_H_4_Cl_2_}*_n_*: C_52.80_H_47.20_Cl_1.60_CoN_8_O_2_S_2_, *M* = 1005.60, pink block, monoclinic, space group *P*2_1_/*c*, *a* = 10.3756(6), *b* = 19.1855(11), *c* = 16.2699(9) Å, *β* = 106.881(3)^o^, *U* = 3099.2(3) Å^3^, *Z* = 2, *D_c_* = 1.078 Mg m^–3^, *μ*(Cu-Kα) = 3.749 mm^−1^, *T* = 123 K. Total 31,013 reflections, 5731 unique, *R*_int_ = 0.033. Refinement of 4672 reflections (282 parameters) with *I* > 2σ (*I*) converged at final *R*1 = 0.1176 (*R*1 all data = 0.1247), *wR*2 = 0.1340 (*wR*2 all data = 0.1341), gof = 0.9888. CCDC 1877183.

{[Co(NCS)_2_(**4**)]·1.6H_2_O·1.2C_6_H_4_Cl_2_}*_n_*: C_65.20_H_56.00_Cl_2.40_CoN_8_O_3.60_S_2_, *M* = 1217.36, pink block, monoclinic, space group *P*2_1_/*c*, *a* = 13.7465(6), *b* = 15.7832(7), *c* = 16.2872(8) Å, *β* = 112.147(2)^o^, *U* = 3273.0(3) Å^3^, *Z* = 2, *D_c_* = 1.235 Mg m^–3^, *μ* (Cu-Kα) = 3.953 mm^−1^, *T* = 123 K. Total 23,855 reflections, 5937 unique, *R*_int_ = 0.035. Refinement of 5459 reflections (313 parameters) with *I* >2σ (*I*) converged at final *R*1 = 0.1460 (*R*1 all data = 0.1460), *wR*2 = 0.1415, gof = 0.9888. CCDC 1877182.

[Co(NCS)_2_(MeOH)_2_(**5**)_2_]·2CHCl_3_·2MeOH: C_108_H_90_Cl_6_CoN_14_O_8_S_2_, *M* = 2047.77, pink block, triclinic, space group *P*–1, *a* = 14.1006(10), *b* = 14.5250(10), *c* = 14.5512(11) Å, *α* =62.394(3), *β* = 89.108(3), *γ* = 64.293(3)^o^, *U* = 2314.5(3) Å^3^, *Z* = 1, *D_c_* = 1.469 Mg m^–3^, *μ*(Cu-Kα) = 4.035 mm^−1^, *T* = 123 K. Total 26,118 reflections, 8388 unique, *R*_int_ = 0.026. Refinement of 7965 reflections (618 parameters) with *I* > 2σ (*I*) converged at final *R*1 = 0.1090 (*R*1 all data = 0.2656), *wR*2 = 0.1117 (*wR*2 all data = 0.2664), gof = 0.9524. CCDC 1877184.

## 3. Results

### 3.1. Synthesis and Characterization of Ligands

**Scheme 4:**
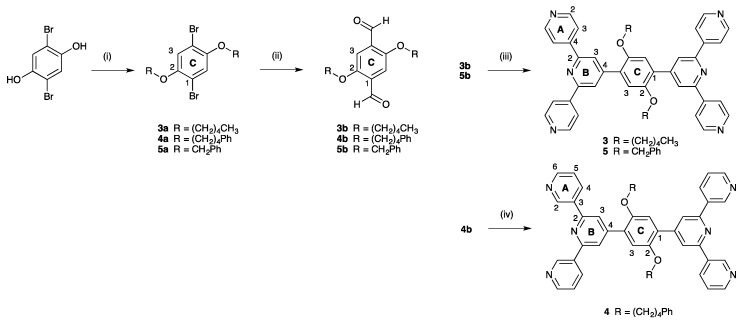
Synthetic routes to **3–5** with numbering for NMR spectroscopic assignments in the experimental section; CH_2_ units in alkyloxy chains are labelled a, b, c… etc. starting at the OCH_2_ unit and the terminal phenyl rings are labelled D. Conditions: (i) RBr or benzyl chloride (see Materials and Methods), anhydrous K_2_CO_3_, dry DMF, 100 °C, 15 h; (ii) *^n^*BuLi, Et_2_O, 0 °C; DMF, warmed to room temperature, 15 h; (iii) 4-acetylpyridine, KOH, EtOH, aqueous NH_3_, room temperature, 15 h; (iv) 3-acetylpyridine, KOH, EtOH, aqueous NH_3_, room temperature, 15 h.

**Figure 1:**
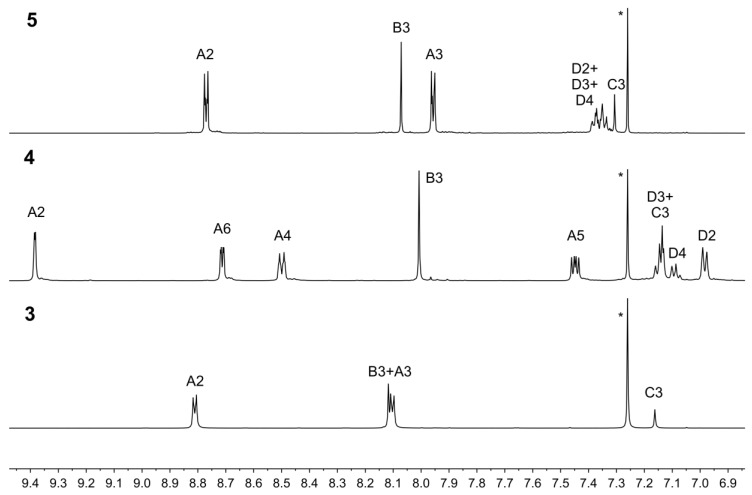
Aromatic regions of the ^1^H NMR spectra of ligands **3**–**5** (500 MHz, CDCl_3_, 298 K). See Scheme 4 for atom labels. * = residual CHCl_3_.

Compounds **3**–**5** were prepared following the one-pot strategy of Wang and Hanan [36] (right-side of Scheme 4), an approach that requires the appropriate dicarbaldehyde. Precursors **3a**–**5a** (Scheme 4) were prepared by treatment of 2,5-dibromohydroquinone with 1-bromopentane, 1-bromo-4-phenylbutane or benzyl chloride under basic conditions. Subsequent lithiation and treatment with DMF followed by addition of aqueous NH_4_Cl yielded the dicarbaldehydes **3b**–**5b.** After work-up, compounds **3**–**5** were isolated as white solids in yields ranging from 18.8 to 37.8%. In the HR-ESI mass spectrum of each compound, the highest-mass peak corresponded to the [M + H]^+^ ion (*m**/**z* = 713.3600, 837.3901 and 753.2967, respectively). For **3** and **5**, this was the base peak, and lower intensity peaks at 357.1841 and 377.1522, respectively, arose from the [M + 2H]^2+^ ion. In the HR-ESI mass spectrum of **4**, the [M + 2H]^2+^ ion gave the base peak (*m*/*z* = 419.1994), and a peak assigned to the [M + 3H]^3+^ ion (*m*/*z* = 279.8021) was also observed. The mass spectra are shown in Figures S2–S4. The ^1^H and ^13^C NMR spectra of the ligands were assigned by COSY, NOESY, HMQC and HMBC methods, and the atom labelling scheme is defined in Scheme 4. NOESY cross peaks between the signals for protons H^A3^ and H^B3^ in **3** and **5** distinguished H^A3^ from H^A2^. Figure 1 displays the aromatic regions of the ^1^H NMR spectra and Figures S5–S10 show full ^1^H and ^13^C NMR spectra. 

### 3.2. Crystal Growth

All crystal growth experiments were carried out under ambient conditions by layering a methanol solution of Co(NCS)_2_ over a solution of the ligand in either 1,2-dichlorobenzene or chloroform (see Materials and methods). Parallel crystal-setups using 1,2-dichlorobenzene or chloroform for each ligand/Co(NCS)_2_ combination were made, and the ratio of Co(NCS)_2_: ligand was either 1:1 or 2:1. For ligands **3** and **4**, pink crystals were harvested after 2–4 weeks, although in the case of **3**, few crystals formed. X-ray quality crystals were only obtained when 1,2-dichlorobenzene was used during crystal growth, and single crystal X-ray diffraction established the assembly of coordination networks (see below). For ligand **5**, crystals were only obtained when using a solution of **5** in chloroform, and X-ray diffraction revealed the unexpected discrete coordination compound [Co(NCS)_2_(MeOH)_2_(**5**)_2_]·2CHCl_3_·2MeOH. 

### 3.3. {[Co(NCS)_2_(**3**)]·0.8C_6_H_4_Cl_2_}_n_ and {[Co(NCS)_2_(**4**)]·1.6H_2_O·1.2C_6_H_4_Cl_2_}_n_

**Figure 2:**
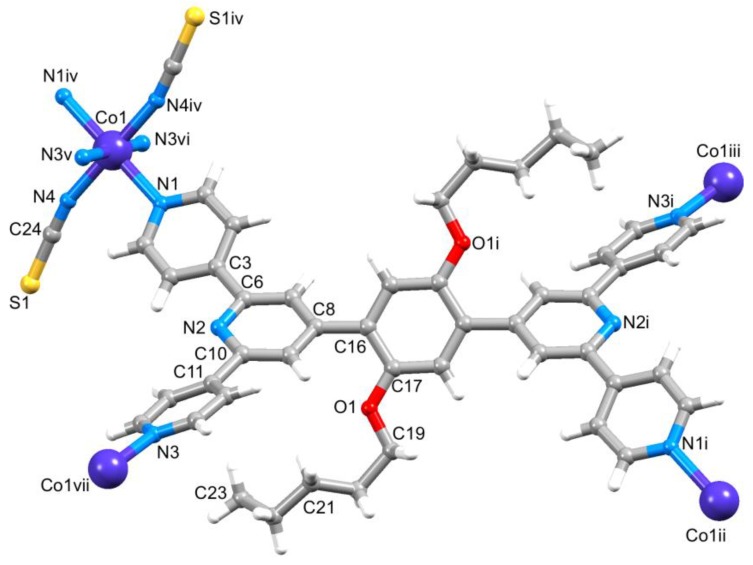
The asymmetric unit in {[Co(NCS)_2_(**3**)]·0.8C_6_H_4_Cl_2_}*_n_* and symmetry-generated atoms to show ligand **3** as a 4-connecting node. Symmetry codes: i = −1 − *x*, 1 – *y*, 1 − *z*; ii = −1 + *x*, *y*, 1 + *z*; iii = −1 + *x*, ^3^/_2_ − *y*, ½ + *z*; iv = −*x*, 1 − *y*, −*z*; v = *x*, ½ − *y*, −^1^/_2_ + *z*; vi = −*x*, ½ + *y*, ½ – *z*; vii = *x*, ½ − *y*, ½ + *z*.

**Figure 3:**
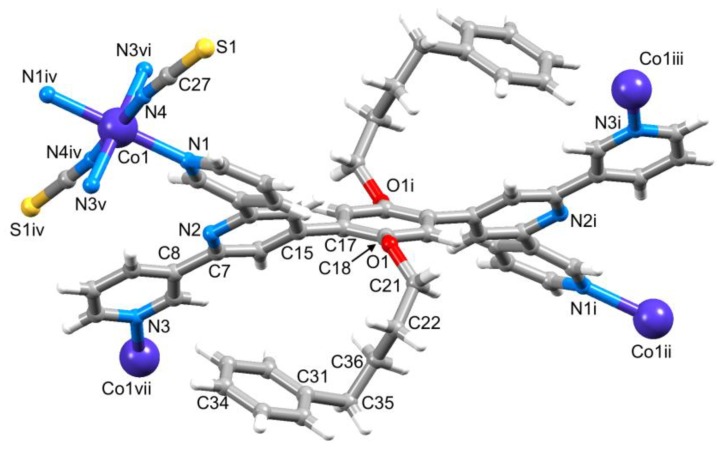
The asymmetric unit in {[Co(NCS)_2_(**4**)]·1.6H_2_O·1.2C_6_H_4_Cl_2_}*_n_* and symmetry-generated atoms to show ligand **4** as a 4-connecting node. Symmetry codes: i = −1 − *x*, 2 − *y*, 1 − *z*; ii = −1 + *x*, 1 + *y*, *z*; iii = −1 − *x*, ½ + *y*, ½ − *z*; iv = −*x*, 1 − *y*, 1 − *z*; v = −*x*, −^1^/_2_ + *y*, ^3^/_2_ − *z*; vi = *x*, ^3^/_2_ − *y*, −^1^/_2_ + *z*; vii = −*x*, ½ + *y*, ^3^/_2_ − *z*.

Single crystal X-ray diffraction confirmed that reactions of Co(NCS)_2_ with **3** and **4** yielded the coordination assemblies {[Co(NCS)_2_(**3**)]·0.8C_6_H_4_Cl_2_}*_n_* and {[Co(NCS)_2_(**4**)]·1.6H_2_O·1.2C_6_H_4_Cl_2_}*_n_*. For the latter, verification that the single crystal was representative of the bulk sample was obtained from powder X-ray diffraction (Figure S1). Both {[Co(NCS)_2_(**3**)]·0.8C_6_H_4_Cl_2_}*_n_* and {[Co(NCS)_2_(**4**)]·1.6H_2_O·1.2C_6_H_4_Cl_2_}*_n_* crystallize in the monoclinic space group *P*2_1_/*c*. The cobalt(II) center in each compound is in a similar six-coordinate environment with *trans*-thiocyanato ligands and nitrogen donors from four different ligands defining the equatorial sites. The asymmetric unit in each structure contains one cobalt atom and half of a ligand **3** or **4**. In {[Co(NCS)_2_(**3**)]·0.8C_6_H_4_Cl_2_}*_n_*, the asymmetric unit contains 0.4 C_6_H_4_Cl_2_, while in {[Co(NCS)_2_(**4**)]·1.6H_2_O·1.2C_6_H_4_Cl_2_}*_n_*, the asymmetric unit has 0.6 C_6_H_4_Cl_2_ disordered over two orientations modelled with equal occupancies. For the compound with ligand **4**, the pyridine ring containing N1 is disordered and was modelled over two sites of occupancies 0.6 and 0.4. The O atom of the 4-phenyl-*n*-butoxy chain is also disordered, and was again modelled over two sites with occupancies 0.6 and 0.4. In the discussion below, we focus only on the major occupancy sites in {[Co(NCS)_2_(**4**)]·1.6H_2_O·1.2C_6_H_4_Cl_2_}*_n_*. Figure 2 and Figure 3 illustrate that each of ligands **3** and **4** binds four cobalt (II) centers, thereby acting as a 4-connecting node. Table 1 gives bond parameters within each cobalt (II) coordination sphere. These parameters and all other bond distances and angles in the structures are unremarkable, and do not warrant further comment.

**Figure 4:**
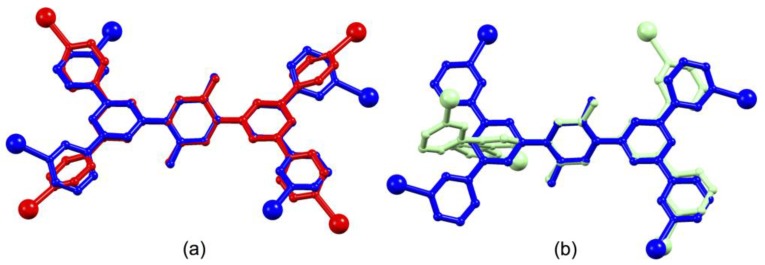
(**a**) Overlay of {Co_4_(**3**)} and {Co_4_(**4**)} units in {[Co(NCS)_2_(**3**)]·0.8C_6_H_4_Cl_2_}*_n_* and {[Co(NCS)_2_(**4**)]·1.6H_2_O·1.2C_6_H_4_Cl_2_}*_n_*; (**b**) Overlay of {Co_4_(**4**)} and {Co_4_(**2a**)} units in {[Co(NCS)_2_(**4**)]·1.6H_2_O·1.2C_6_H_4_Cl_2_}*_n_* (this work) and {[Co(NCS)_2_(**2a**)]·4CHCl_3_}*_n_* [20]. In each overlay, the atoms of the central phenylene ring superimposed. For clarity, only the O atoms of the alkyloxy chains are shown.

**Figure 5:**
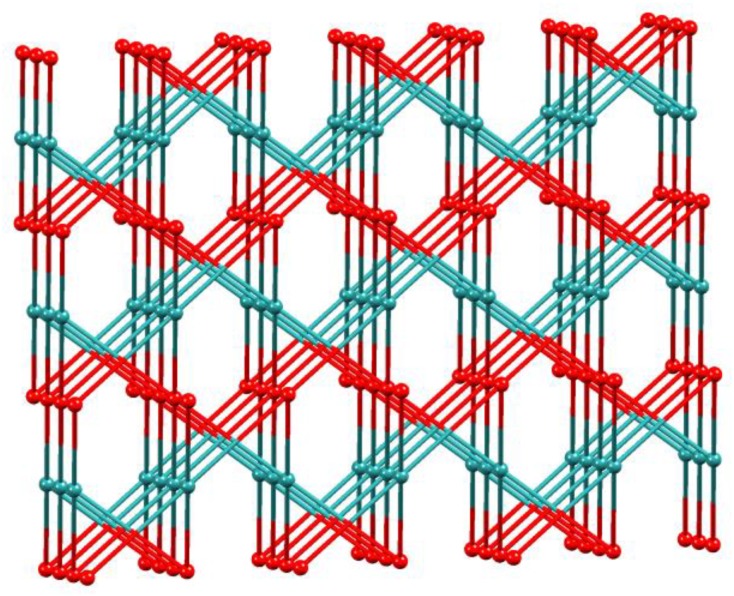
Topological representation of the 3D net in {[Co(NCS)_2_(**4**)]·1.6H_2_O·1.2C_6_H_4_Cl_2_}*_n_* generated using Mercury [34,35] with 4-connecting cobalt and ligand nodes. The net in {[Co(NCS)_2_(**3**)]·0.8C_6_H_4_Cl_2_}*_n_* is the same (see Figure S12).

The dihedral angles between the planes through adjacent aromatic rings in coordinated **3** and **4** are given in the first two entries in Table 2. The twist of the ring with N2 with respect to the phenylene ring in each compound is typical of covalently bonded arene rings and minimizes H…H repulsions. There are small differences in the conformations of the 4,2′:6′,4″- and 3,2′:6′,3″-tpy units in coordinated **3** and **4**, as displayed in Figure 4a in the overlay of the {Co_4_(**3**)} and {Co_4_(**4**)} units. However, both **3** and **4** function as planar 4-connecting nodes and, despite the isomeric donor sets, each propagates into a {6^5^.8} *cds* net (Figure 5 and Figure S12) in which half of the adjacent nodes are mutually perpendicular and half are coplanar [26]. This resembles the 3D net observed in {[Co(NCS)_2_(**1d**)]·2C_6_H_4_Cl_2_}*_n_* [28] and, for comparison, the corresponding dihedral angles for this structure are given in Table 1. In fact, the unit cell dimensions of {[Co(NCS)_2_(**3**)]·0.8C_6_H_4_Cl_2_}*_n_* and {[Co(NCS)_2_(**1d**)]·2C_6_H_4_Cl_2_}*_n_* (both in the space group *P*2_1_/*c*) are very similar (*a* = 10.3756(6), *b* = 19.1855(11), *c* = 16.2699(9) Å, *β* = 106.881(3)^o^, *U* = 3099.2(3) Å^3^ versus *a* = 10.2136(9), *b* = 19.3452(17), *c* = 16.2214(15) Å, *β* = 107.027(3)^o^, *U* = 3064.6(5) Å^3^), revealing that the network can accommodate either *n*-pentyloxy or *n*-propoxy chains without significant perturbation (Figure S11). 

The phenyl groups in the 4-phenylbutoxy chains in {[Co(NCS)_2_(**4**)]·1.6H_2_O·1.2C_6_H_4_Cl_2_}*_n_* do not play a critical role in the assembly process and are not involved in π-stacking interactions. This is in contrast to the 3-phenylpropoxy groups in [Zn_2_Br_4_(**1c**)**^.^**H_2_O]*_n_* (see introduction) [25]. Instead, the 1,2-dichlorobenzene solvate in {[Co(NCS)_2_(**4**)]·1.6H_2_O·1.2C_6_H_4_Cl_2_}*_n_* engages in a face-to-face π-interaction with the pyridine ring containing atom N3 (Figure S13). Pertinent parameters are centroid…centroid and centroid…pyridine-plane separations of 3.74 and 3.49 Å, respectively, and an angle between the ring planes of 9.7°. However, since the solvent site is only partially occupied, one must be cautious in over-discussing both these interactions and the Cl…H–C contacts shown in Figure S13.

It is instructive to compare the structures of {[Co(NCS)_2_(**4**)]·1.6H_2_O·1.2C_6_H_4_Cl_2_}*_n_* (this work) and {[Co(NCS)_2_(**2a**)]·4CHCl_3_}*_n_* [20]. These compounds both have 3,2′:6′,3″-tpy donor sets, but differ in the nature of the alkyloxy chain (4-phenylbutoxy *versus n*-octyloxy). A second difference is the solvent system for crystal growth (1,2-dichlorobenzene *versus* chloroform). Figure 4b illustrates an overlay of the {Co_4_(**4**)} and {Co_4_(**2a**)} units in {[Co(NCS)_2_(**4**)]·1.6H_2_O·1.2C_6_H_4_Cl_2_}*_n_* and {[Co(NCS)_2_(**2a**)]·4CHCl_3_}*_n_*, and reveals a significant difference in ligand conformation. This arises from the larger dihedral angles between the 3,2′:6′,3″-tpy ring with N2 and the phenylene ring (Table 1). As previously described, {[Co(NCS)_2_(**2a**)]·4CHCl_3_}*_n_* possesses a {4^2^.8^4^} *lvt* net. It is now clear that the assembly of the *lvt* net is not simply a consequence of introducing the 3,2′:6′,3″-tpy in place of the 4,2′:6′,4″-tpy domain. Unfortunately, we have not been able to obtain good quality X-ray diffraction data from crystals grown from a combination of Co(NCS)_2_ with **1b** (Scheme 2) and are unable, therefore, to verify whether the switch from the *cds* to *lvt* net is a consequence of the steric demands of the long *n*-octyl chain.

### 3.4. [Co(NCS)_2_(MeOH)_2_(**5**)_2_]·2CHCl_3_·2MeOH

**Figure 6:**
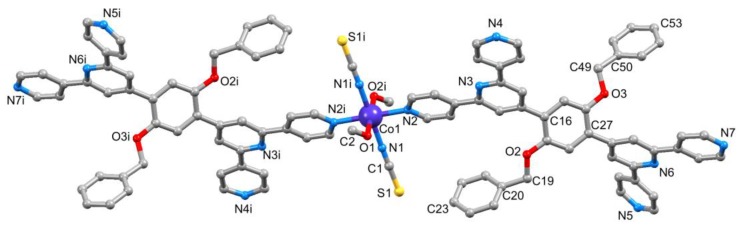
Molecular structure of [Co(NCS)_2_(MeOH)_2_(**5**)_2_]·2CHCl_3_·2MeOH with solvent molecules and H atoms omitted. Selected bond distances and angles: Co1–O1 = 2.138(3), Co1–N1 = 2.090(4), Co1–N2 = 2.167(3) Å, O1–Co1–N2 = 86.80(12), O1–Co1–N1 = 88.39(15), N2–Co1–N1 = 88.06(13), N1–Co1–N2^i^ = 91.94(13), N1–Co1–O1^i^ = 91.61(15), O1–Co1–N2^i^ = 93.20(12)°. Symmetry code i = 1 − *x*, 3 − *y*, −*z*.

**Scheme 5:**
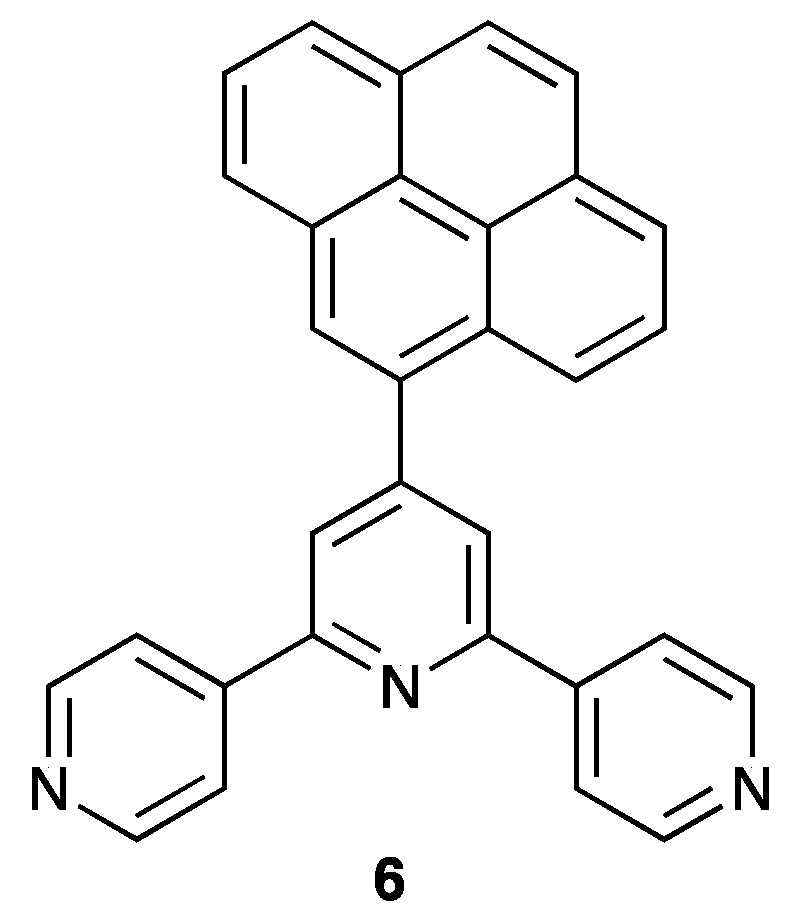
The structure of ligand **6** from reference [44].

**Figure 7:**
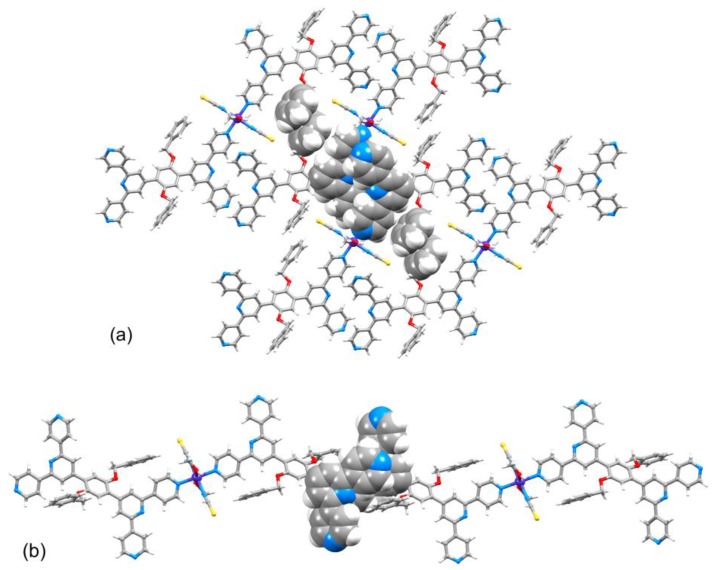
Packing interactions in [Co(NCS)_2_(MeOH)_2_(**5**)_2_]·2CHCl_3_·2MeOH (solvent molecules omitted) (**a**) between pairs of phenyl rings containing C20 and C20^ii^ (symmetry code ii = 2 − *x*, 2 − *y*, 1 − *z*) and pairs of 4,2′:6′,4″-tpy units containing N2/N4 and N2^iii^/N4^iii^ (symmetry code iii = 1 − *x*, 2 − *y*, −*z*) and (**b**) pairs of 4,2′:6′,4″-tpy units containing N6/N7 and N6^iv^/N7^iv^ (symmetry code iv = 2 − *x*, −*y*, 2 − *z*).

The discussion above demonstrates that consistent networks can be obtained with a combination of Co(NCS)_2_ and tetratopic ligands incorporating either 3,2′:6′,3″- or 4,2′:6′,4″-tpy metal-binding domains. The observations suggest that the nature of the alkyloxy substituents, rather than the choice of tpy isomer, may be the critical factor in determining the outcome of the coordination assembly. On going from ligand **3** to **5**, we gain the potential for π-stacking without significantly lengthening the alkyloxy chain. Layering an MeOH solution of Co(NCS)_2_ over a CHCl_3_ solution of **5** produced only a few crystals, and there was insufficient material to obtain powder XRD data for the bulk sample. Single crystal X-ray diffraction revealed the unexpected formation of the discrete molecular coordination compound [Co(NCS)_2_(MeOH)_2_(**5**)_2_]·2CHCl_3_·2MeOH. The compound crystallizes in the triclinic space group *P*–1, and the centrosymmetric structure is shown in Figure 6 with selected bond parameters given in the figure caption. Each ligand **5** acts as a monodentate *N*-donor ligand and MeOH molecules complete the octahedral coordination sphere of Co1. While {Co(NCS)_2_(MeOH)_2_(py)_2_} (py = pyridine) motifs are well established [37,38,39,40,41,42], there is only one example in the CSD (v. 5.39 with updates [43]) featuring a monotopic 4,2′:6′,4″-tpy ligand **6** (Scheme 5) [44]. In this work, crystal growth by layering a MeOH/Co(NCS)_2_ solution over a solution of **6** in MeOH/CH_2_Cl_2_ with an intermediate MeOH/CH_2_Cl_2_ layer resulted in the formation of [Co(NCS)_2_(MeOH)_2_(**6**)_2_]. The preference for this discrete molecular assembly was attributed to the introduction of the sterically demanding pyrenyl group which is involved in efficient intermolecular pyrene…4,2′:6′,4″-tpy π-stacking interactions [44]. In the case of [Co(NCS)_2_(MeOH)_2_(**5**)_2_]·2CHCl_3_·2MeOH, packing interactions (Figure 7) involve centrosymmeric pairs of phenyl rings containing C20 and C20^ii^ (symmetry code ii = 2 − *x*, 2 − *y*, 1 − *z*), pairs of 4,2′:6′,4″-tpy units containing N2/N4 and N2^iii^/N4^iii^ (symmetry code iii = 1 − *x*, 2 − *y*, −*z*), and pairs of 4,2′:6′,4″-tpy units containing N6/N7 and N6^iv^/N7^iv^ (symmetry code iv = 2 − *x*, −*y*, 2 − *z*). The first two sets of interactions lock together four molecules (Figure 7a). For the phenyl…phenyl π-stacking interaction, the separations of the ring planes and centroids are 3.35 and 3.65 Å, respectively. The coordinated 4,2′:6′,4″-tpy unit with N2/N3/N4 is virtually planar (dihedral angles between the ring planes = 7.4 and 6.0°), and 4,2′:6′,4″-tpys with N2/N4 and N2^iii^/N4^iii^ engage in a π-stacking interaction (Figure 7a) with an angle between the ring planes of 6.0° and centroid…centroid distance of 3.60 Å. For the non-coordinated 4,2′:6′,4″-tpy unit, the dihedral angles between pairs of rings with N5/N6 and N6/N7 are 24.1 and 3.2°, respectively. Figure 7b depicts the face-to-face π-stacking interaction pairs of 4,2′:6′,4″-tpys containing N6/N7 and N6^iv^/N7^iv^ (centroid…centroid = 3.83 Å). The overall packing in the lattice is, therefore, dominated by efficient π-stacking [45], but, because of the low yield of crystals, we are unable to say whether this leads to a preference for a discrete molecular structure in which three of the four potential metal binding sites of ligand **5** remain non-coordinated, or whether the isolation of crystals of [Co(NCS)_2_(MeOH)_2_(**5**)_2_]·2CHCl_3_·2MeOH is serendipitous. 

## 4. Conclusions

We have prepared three new ligands containing two 4,2′:6′,4″-tpy or two 3,2′:6′,3″-tpy metal-binding domains with the expectation that they would function as 4-connecting nodes in coordination networks. The ligands possess different alkyloxy functionalities attached to the central phenylene spacer: *n*-pentyloxy in **3**, 4-phenylbutoxy in **4**, benzyloxy in **5**. Reactions with Co(NCS)_2_ under ambient conditions with MeOH and 1,2-C_6_H_4_Cl_2_ as solvents resulted in the formation of {[Co(NCS)_2_(**3**)]·0.8C_6_H_4_Cl_2_}*_n_* and {[Co(NCS)_2_(**4**)]·1.6H_2_O·1.2C_6_H_4_Cl_2_}*_n_*, both of which exhibit 3D *cds* nets. These data taken along with previously reported related structures suggests that the assembly of a particular net (*cds* or *lvt*) with 4-connecting Co and bis(tpy) ligands is independent of the choice of 4,2′:6′,4″- or 3,2′:6′,3″-tpy. The role of the alkyloxy substituent appears to be more significant. The combination of Co(NCS)_2_ with ligand **5** resulted in the formation of the discrete molecular complex [Co(NCS)_2_(MeOH)_2_(**5**)_2_]·2CHCl_3_·2MeOH in which **5** behaves as a monodentate ligand. The pendant phenyls and both coordinated and non-coordinated 4,2′:6′,4″-tpy units engage in efficient π-stacking interactions. However, we are unable to identify whether the isolation of the mononuclear complex is preferred over network assembly, or is a serendipitous result. Further systematic investigations are needed in order to delineate the assembly algorithms in these systems.

## Figures and Tables

**Table 1:** Important bond parameters in {[Co(NCS)_2_(**3**)]·0.8C_6_H_4_Cl_2_}*_n_* and {[Co(NCS)_2_(**4**)]·1.6H_2_O·1.2C_6_H_4_Cl_2_}*_n_*.

Bond Parameter	{[Co(NCS)_2_(3)]·0.8C_6_H_4_Cl_2_}*_n_*	{[Co(NCS)_2_(4)]·1.6H_2_O·1.2C_6_H_4_Cl_2_}*_n_*
Distance/Å		
Co1–N1	2.208 (2)	2.238 (4)
Co1–N4	2.062 (3)	2.065 (4)
Co1–N3v	2.179 (2)	2.169 (4)
Angle/deg		
N1–Co1–N4	90.18 (10)	88.67 (15)
N3v–Co1–N1	84.73 (9)	93.81 (17)
N3v–Co1–N4	90.20 (11)	90.32 (16)
N3vi–Co1–N1	95.27 (9)	86.19 (17)
N3vi–Co1–N4	89.80 (11)	89.68 (16)
N1iv–Co1–N4	89.82 (10)	91.33 (15)

**Table 2:** Dihedral angles between pairs of bonded aromatic rings in {[Co(NCS)_2_(**3**)]·0.8C_6_H_4_Cl_2_}*_n_* and {[Co(NCS)_2_(**4**)]·1.6H_2_O·1.2C_6_H_4_Cl_2_}*_n_*, and in {[Co(NCS)_2_(**1d**)]·2C_6_H_4_Cl_2_}*_n_* [28] and {[Co(NCS)_2_(**2a**)]·4CHCl_3_}*_n_* [20].

Compound	Dihedral Angle between Planes/Deg		
	Ring with N1/Ring with N2	Ring with N2/Ring with N3	Ring with N2/Phenylene Ring
{[Co(NCS)_2_(**3**)]·0.8C_6_H_4_Cl_2_}*_n_*	25.3	30.7	40.2
{[Co(NCS)_2_(**4**)]·1.6H_2_O·1.2C_6_H_4_Cl_2_}*_n_*	34.6	6.4	44.3
{[Co(NCS)_2_(**1d**)]·2C_6_H_4_Cl_2_}*_n_*	19.5	31.3	40.5
{[Co(NCS)_2_(**2a**)]·4CHCl_3_}*_n_*	29.9; 22.7	12.7; 12.2	58.5; 55.9

## Supplementary Materials

The following are available online at http://www.mdpi.com/2073-4360/10/12/1369/s1, Figure S1: Powder diffraction data for the bulk sample of {[Co(NCS)_2_(**4**)]·1.6H_2_O·1.2C_6_H_4_Cl_2_}*_n_*. Figures S2–S4: High resolution electrospray (HR-ESI) mass spectra of **3**, **4** and **5**. Figures S5–S10: ^1^H and ^13^C NMR spectra of **3**, **4** and **5**. Figure S11: Overlay of {Co_4_(**3**)} and {Co_4_(**1d**)} units in {[Co(NCS)_2_(**3**)]·0.8C_6_H_4_Cl_2_}*_n_* and {[Co(NCS)_2_(**1d**)]·2C_6_H_4_Cl_2_}*_n_*. Figure S12: Topological representation of the 3D net in {[Co(NCS)_2_(**3**)]·0.8C_6_H_4_Cl_2_}*_n_*. Figure S13: Close contacts involving the 1,2-dichlorobenzene molecule in {[Co(NCS)_2_(**4**)]·1.6H_2_O·1.2C_6_H_4_Cl_2_}*_n_*.
