# Supplementary material for: Sometimes the Same, Sometimes Different: Understanding Self-Assembly Algorithms in Coordination Networks
Source: Polymers (Basel). 2018 Dec 11;10(12):1369. doi: 10.3390/polym10121369 (PMC6401813; doi:10.3390/polym10121369)
Supplement: Supplementary file 1 [file polymers-10-01369-s001.pdf]

Supplementary data for:

Sometimes the same, sometimes different: understanding self-assembly algorithms in coordination networks

Y. Maximilian Klein [1], Alessandro Prescimone [1], Mariia Karpacheva [1], Edwin C. Constable [1] and Catherine E. Housecroft [1,*]

[1] Department of Chemistry, University of Basel, BPR 1096, Mattenstrasse 24a, 4058-Basel, Switzerland
* Correspondence: catherine.housecroft@unibas.ch; Tel.: +41-61-207-1008

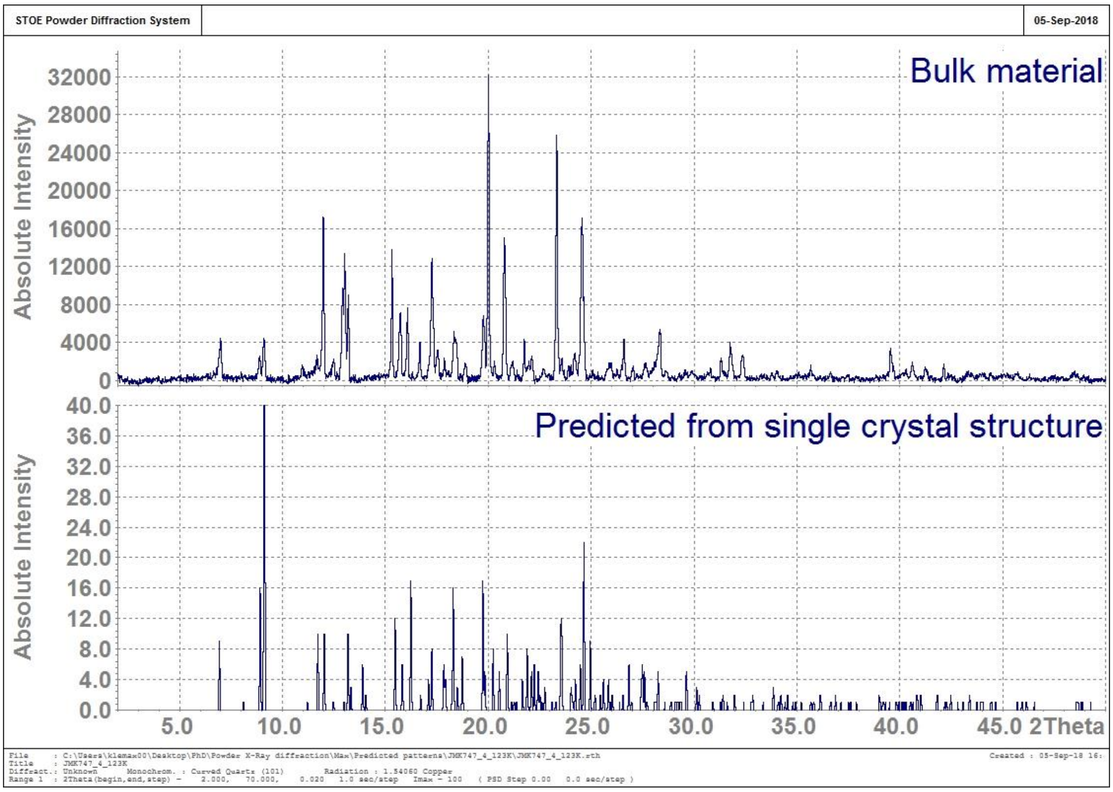

Fig. S1. Powder diffraction data for the bulk sample after removal of crystals from mother liquor compared to the powder pattern predicted from the single crystal structure of $\{[Co(NCS)_2(\mathbf{4})]\cdot 1.6H_2O\cdot 1.2C_6H_4Cl_2\}_n$.

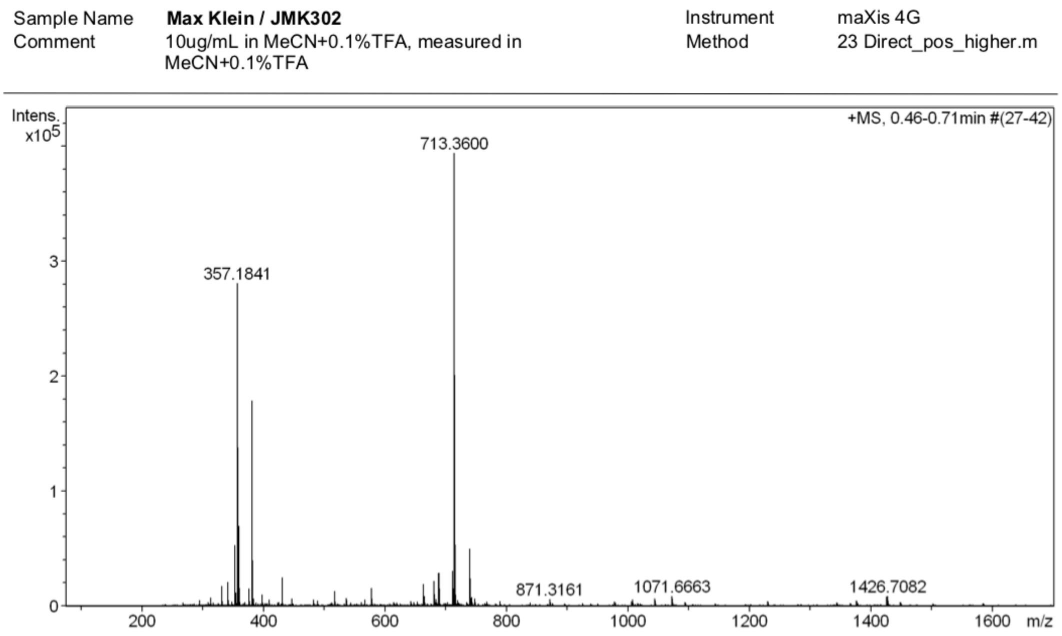

Fig. S2. High resolution electrospray (HR-ESI) mass spectrum of **3**.

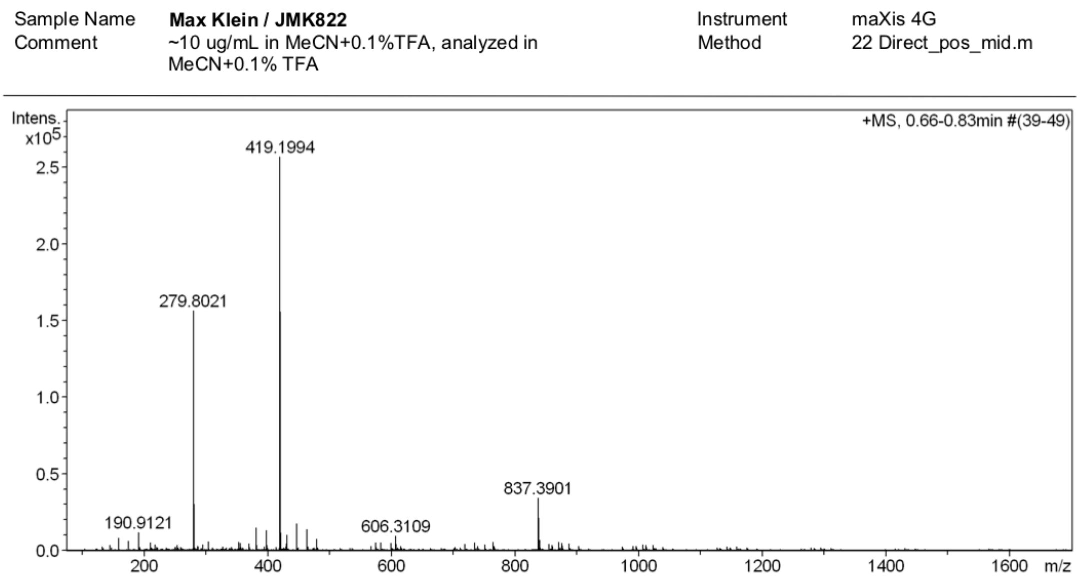

Fig. S3. High resolution electrospray (HR-ESI) mass spectrum of **4**.

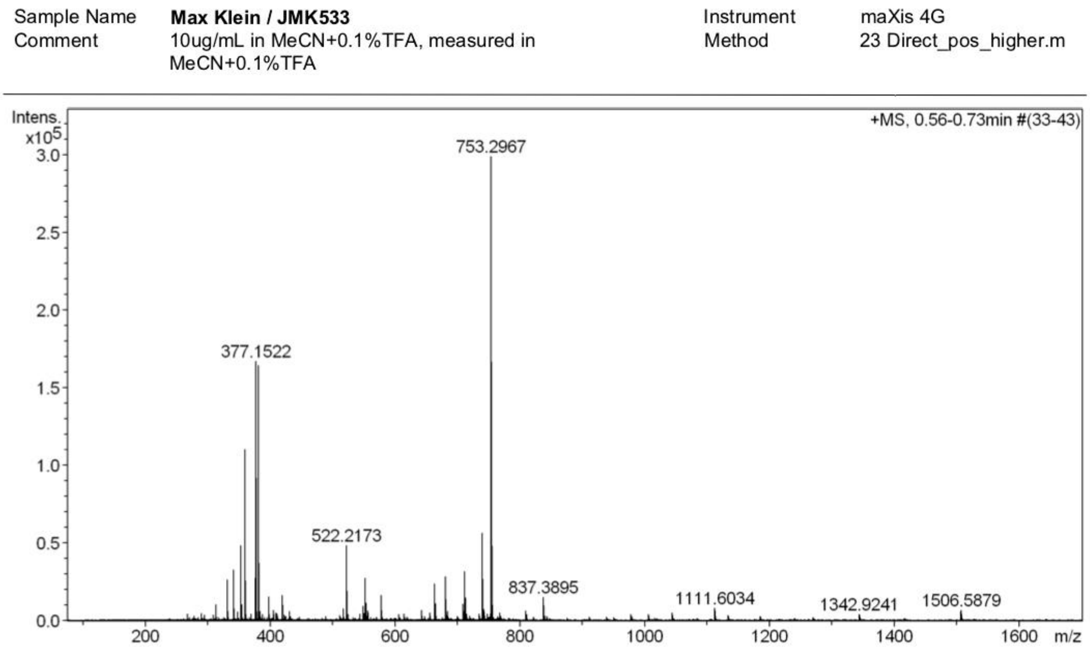

Fig. S4. High resolution electrospray (HR-ESI) mass spectrum of **5**.

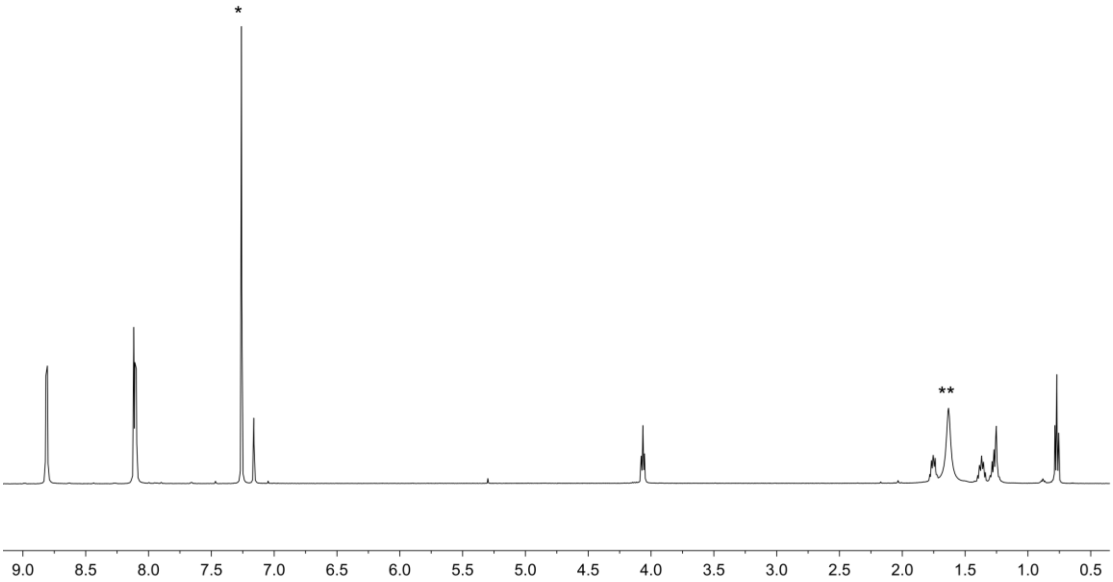

Fig. S5. ^{1}H NMR spectrum of **3** (500 MHz, $CDCl_3$, 298 K). * = residual $CHCl_3$; ** = H_2O.

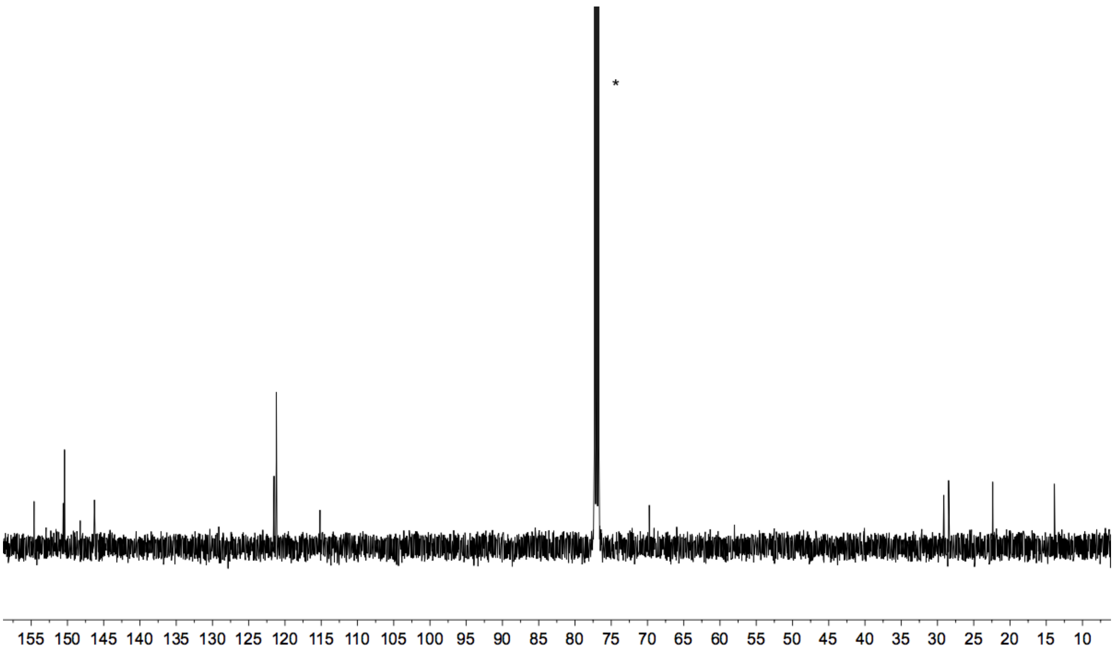

Fig. S6. ^{13}C NMR spectrum of **3** (126 MHz, $CDCl_3$, 298 K). * = $CDCl_3$.

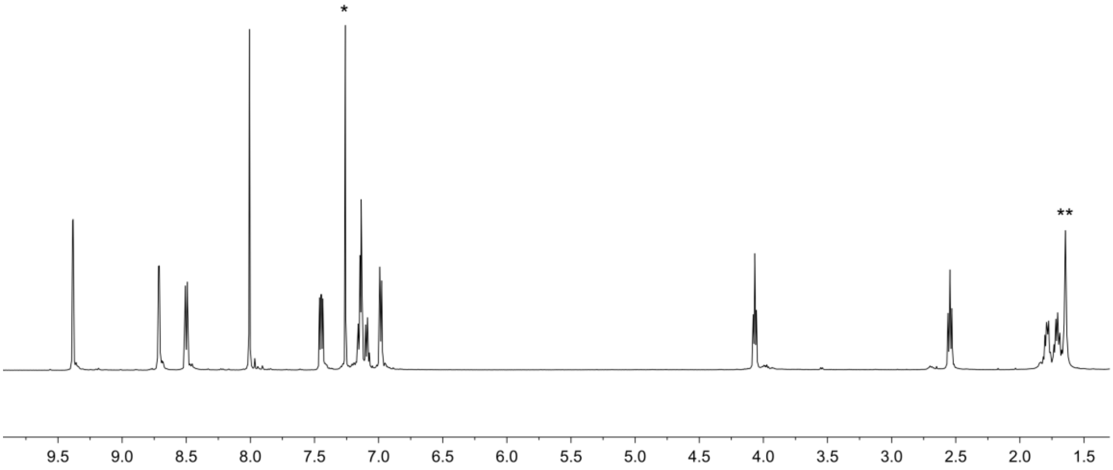

Fig. S7. ^{1}H NMR spectrum of **4** (500 MHz, $CDCl_3$, 298 K). * = residual $CHCl_3$; ** = H_2O.

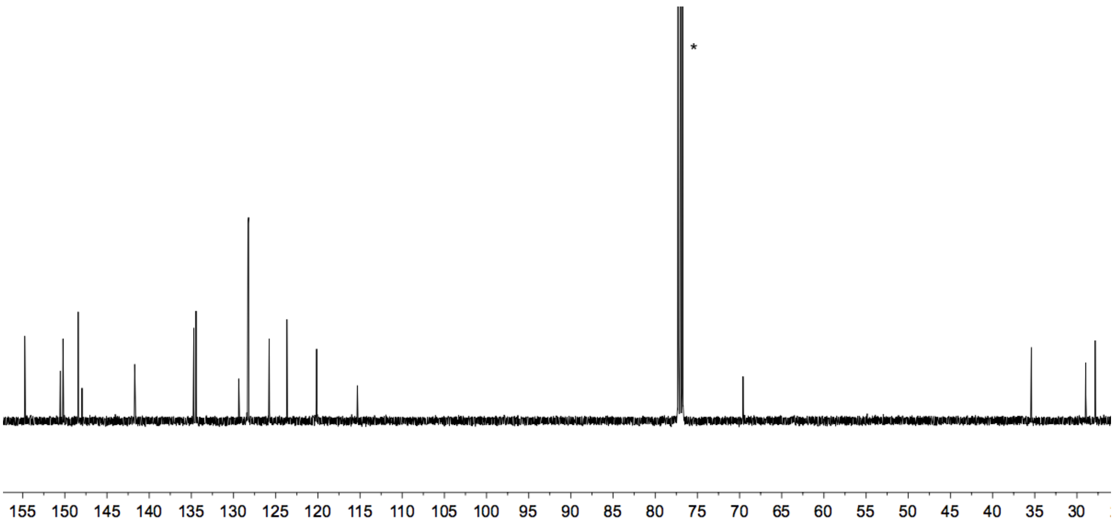

Fig. S8. ^{13}C NMR spectrum of **4** (126 MHz, $CDCl_3$, 298 K). * = $CDCl_3$.

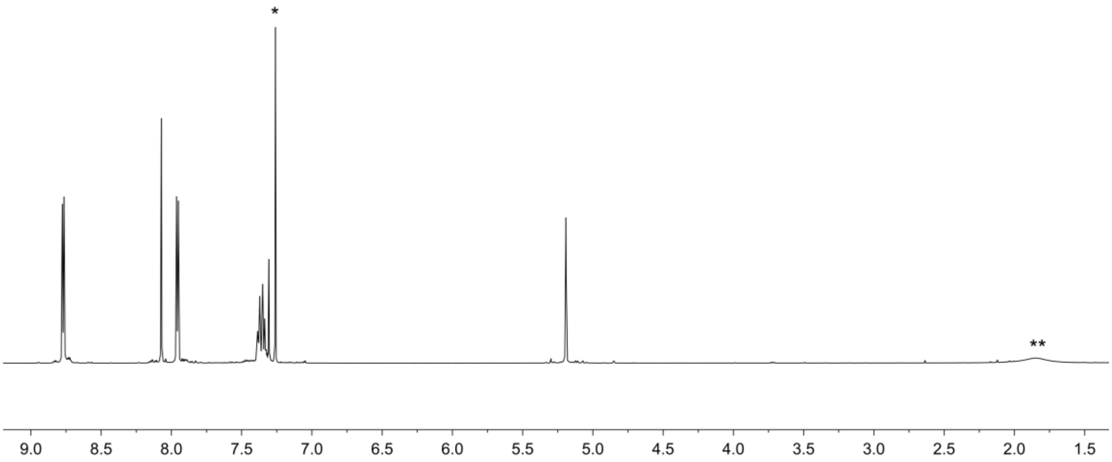

Fig. S9. ^{1}H NMR spectrum of **5** (500 MHz, $CDCl_3$, 298 K). * = residual $CHCl_3$; ** = H_2O.

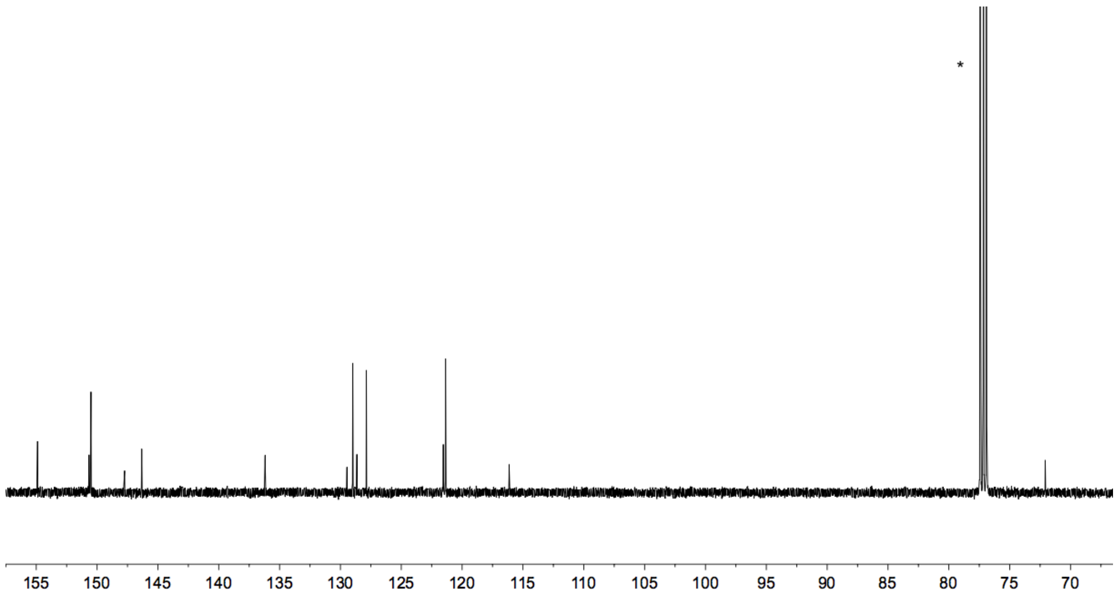

Fig. S10. ^{13}C NMR spectrum of **5** (126 MHz, $CDCl_3$, 298 K). * = $CDCl_3$.

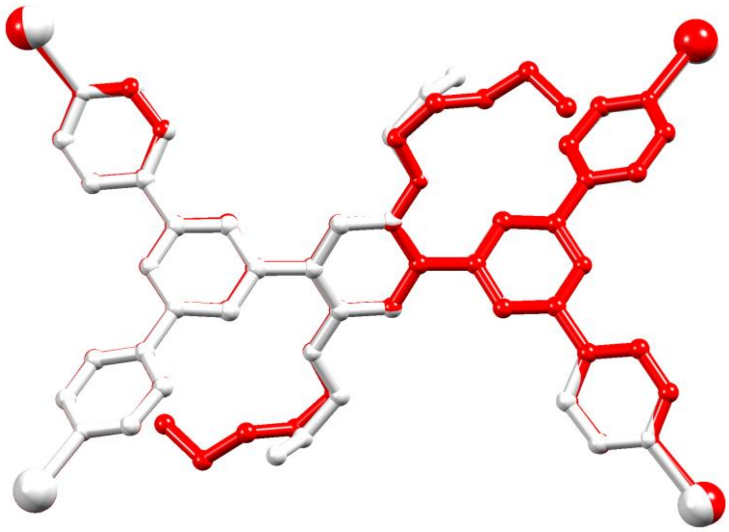

Fig. S11. Overlay of $\{Co_4(\mathbf{3})\}$ (red) and $\{Co_4(\mathbf{1d})\}$ (silver) units in $\{[Co(NCS)_2(\mathbf{3})]\cdot 0.8C_6H_4Cl_2\}_n$ and $\{[Co(NCS)_2(\mathbf{1d})]\cdot 2C_6H_4Cl_2\}_n$ [ref. 28]. In each overlay, the atoms of the central phenylene ring superimposed.

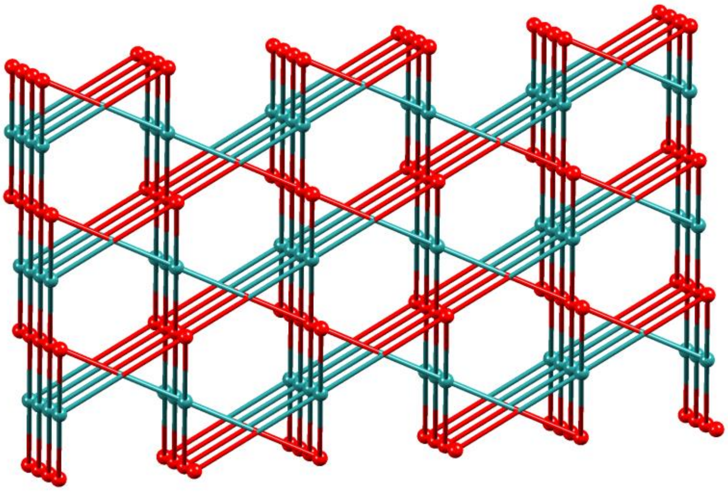

Fig. S12. Topological representation of the 3D net in $\{[Co(NCS)_2(\mathbf{3})]\cdot 0.8C_6H_4Cl_2\}_n$ generated using Mercury [34,35] with 4-connecting cobalt and ligand nodes.

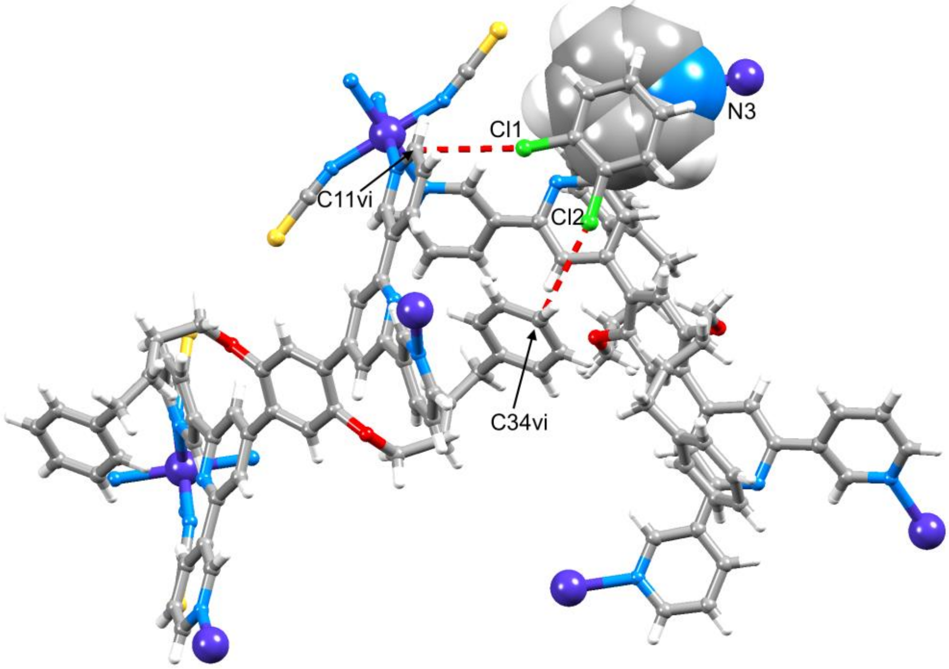

Fig. S13. Close contacts involving the 1,2-dichlorobenzene molecule in $\{[Co(NCS)_2(\mathbf{4})]\cdot 1.6H_2O\cdot 1.2C_6H_4Cl_2\}_n$. Symmetry code vi = x, $^3/_2{-}y$, $-^1/_2{+}z$ (see Fig. 3). Distances: Cl1…C11vi = 3.43, Cl2…C34vi = 3.42 Å. Corresponding Cl…H separations are 3.28 and 2.91 Å. See text for discussion of the π-stacking interaction.
